# Inflammation, oxidative stress, insulin resistance, and hypertension as mediators for adverse effects of obesity on the brain: A review

**DOI:** 10.37796/2211-8039.1174

**Published:** 2021-12-01

**Authors:** Mahnaz Ghowsi, Farshad Qalekhani, Mohammad Hosein Farzaei, Fariba Mahmudii, Namdar Yousofvand, Tanuj Joshi

**Affiliations:** a Department of Biology, Faculty of Sciences, Razi University, Kermanshah, Iran; b Pharmaceutical Sciences Research Center, Health Institute, Kermanshah University of Medical Sciences, Kermanshah, Iran; c Department of Biology, Faculty of sciences, University of Mohaghegh Ardabili, Ardabil, Iran; d Department of Pharmaceutical Sciences, Bhimtal, Kumaun University, Nainital, Uttarakhand, India

**Keywords:** Obesity, Brain damage, Inflammation, Oxidative stress, Insulin resistance, Hypertension

## Abstract

Nowadays, the incidence of obesity is a global challenge and it is estimated that the total number of overweight and obese adults will increase up to 1.35 billion by 2030. Evidence obtained from clinical and experimental studies shows that obesity may be associated with cognitive performance and executive function impairments. Considering various evidence for the poor episodic memory tasks and verbal learning as well as the destruction of cortical gray matter in the obese individuals, here, we collected some causal pathways for contribution of inflammation, oxidative stress, insulin resistance, and hypertension in the development of brain disorders in obesity. The present study focuses on the providing an overview of the some negative effects of obesity on the brain. Different evidence mentioned in this review has thrown light on the obesity-associated complications which may predispose obese people to brain damage, dementia, and Alzheimer’s disease.

## 1. Introduction

Obesity is a prevalent disorder affecting children, adolescents and adults worldwide. World Health Organization (WHO) has estimated that 36.9% of male and 38.0% of female adults are obese and overweight throughout the world. Also, there are about 500 million obese and 2 billion overweight individuals worldwide [1]. The potential association of obesity with many health problems, such as susceptibility to metabolic syndrome, insulin resistance (IR), type 2 diabetes mellitus (T2DM), cardiovascular disease, hypertension, dyslipidemia and cancers, is known [2,3].

Recently, some evidence has shown that there is a negative relationship between the increase in the body weight, body mass index (BMI) or waist circumference and cognitive and executive functions [4]. Dementia is a clinical syndrome in which mental functions such as memory, language, daily life activities and psychosocial activities are progressively disturbed. Epidemiologic studies also indicate that there is a link between obesity due to fat consumption and an increased risk of Alzheimer’s disease (AD), a subtype of dementia [5,6]. There is also a relationship between obesity and the increased BMI, and whole-brain atrophy [7] and the degradation of cortical gray matter [8,9]; the higher the waist-to-hip ratio, the less the hippocampal volume. People with a larger sagittal abdomen are more likely to develop dementia [10,11]; dementia was seen following obesity (BMI>30 kg/m^2^) and overweight (BMI>25–30 kg/m^2^) at midlife [12]. Especially, mid-life central obesity is known as a risk factor for future dementia [11]; the results of a meta-analysis study revealed that obesity increased the AD risk in the elderly subjects [13].

The hippocampus is involved in the process of the feeding behavior, as well as the modulation of memory; its damage may impair learning, anterograde and retrograde memory [14,15]. Also, the amygdala is a critical area involved in learning the taste of food, guiding the appetitive behavior [16]. Evaluation of the brain structure in 527 20–87 year-old subjects, based on the analysis of the cross-sectional magnetic resonance images, showed that the degree of atrophy in the cerebral white-matter volume of overweight and obese adults was greater than that of the lean subjects [17]. Also, the results obtained by magnetic resonance imaging (MRI) have revealed that the volume of the amygdala and hippocampus is increased in obese individuals [16]; however, the mechanisms behind this alteration remain unknown. The memory and cognitive impairments associated with obesity, possibly due to inflammation, gliosis and decreased gray matter of the hippocampus, may lead to an increase in the food intake by obese individuals because they are unable to inhibit the activation of food-related memories and the rewarding consequences of eating [18]. Besides this, in the studies on obese humans and experimental models, inflammation and neuronal injury in the hypothalamus have been observed [19].

**Fig. 1:**
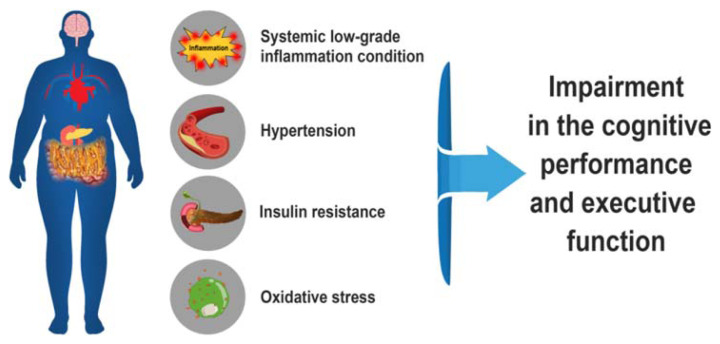
A diagram representing some consequences of obesity on the brain.

All efforts concerning obesity management have been focused on controlling chronic obesity-related diseases like diabetes mellitus and cardiovascular disease; however, other complications associated with obesity and their influence on the brain function should also be taken into account. The present review article, therefore, aims to highlight some potential mechanisms and factors that may affect the brain function over time in obesity. Here, we have summarized the current knowledge to show the adverse effects of oxidative stress, inflammation, IR and hypertension associated with obesity on the brain, as can be seen in Fig. 1.

## 2. Methodology

The authors searched the electronic literature for the English-language articles; these were identified by a series of Google scholar, PubMed, Web of Sciences and Scopus searches using keywords such as obesity, brain, inflammation, oxidative stress, IR, hypertension or their equivalents, individually and/or in different combinations published before September 2020; the goal was to understand some potential mechanisms involved in the impairment of the brain by obesity.

## 3. Results and discussion

### 3.1. Obesity: a low-grade inflammatory condition

Recent studies have considered obesity as a systemic and subclinical low-grade inflammatory condition. Adipose tissue contains adipocytes, fibroblasts, fibroblastic pre-adipocytes, and endothelial and immune cells that secrete hormones and cytokines [3]. The association between the presence of the excess adipose tissue, especially the increased white fat, and chronic inflammation, IR and the levels of the endogenous sex steroids, leptin, visfatin and plasminogen activator inhibitor-1 (PAI-1) is well known [20]. Adipocytes can induce the production of reactive oxygen species (ROS) by the activation of immune cells; this, in turn, can augment the irregular production of adipokines and the inflammatory status [3]. Also, it has been demonstrated that the health risk of the increase in the intra-abdominal fat depot (visceral or central obesity) is greater than that of the subcutaneous accumulation of the excess fat (peripheral obesity). Obesity, particularly central obesity, may be a predictive condition for IR and T2DM, which are among the important underlying factors for inducing inflammation and development of dementia [2].

#### 3.1.1. Effects of inflammation on brain neurogenesis

During neurogenesis, neuroprogenitor cells proliferate and differentiate into neurons, astrocytes and oligodendrocytes. Some studies have shown that obesity may have impact on neurogenesis. In this regard, a study of mice showed that a diet high in fat impaired the neurogenic cells in the hypothalamus by the activation of the NF-kB pathway. The high concentrations of cytokines in the bloodstream may influence neurogenesis; this is because in some studies, the differentiation of neuroproggenitor cells in the presence of cytokines like interleukin (IL)-1β, tumor necrosis factor-alpha (TNF-α) and IL-6 has been shown to reduce their neuronal markers, forcing them to differentiate into a glial phenotype [21,22].

#### 3.1.2. Effects of neuroinflammation on the feeding behavior

The effects of neuroinflammation resulting from obesity are seen in the hypothalamus, amygdala, hippocampus, cortex and cerebellum [23,24]. Obesity caused by overconsuming high fat foods interrupts the anorexigenic and thermogenic signals produced by the hormones leptin and insulin, inducing inflammation in the hypothalamic areas which control the feeding behavior in the animals. So, inflammation may impair neural circuits involved in adjusting the balance between energy intake and energy expenditure [25]. After eating high-fat foods in mice, the expression of the inflammatory markers Il-6 and TNF-α, orexigenic neuropeptides OReXine and agouti-related peptide (AgRP) was increased; also, the inflammation in the hypothalamus induced by the consumption of high-fat diets was associated with the glial reaction. In the long, medium or short term, the increased lipid content in the diet caused inflammation in the hypothalamus; microglial cells and astrocytes are involved in this inflammation. The disruption of the hypothalamic functions by these inflammatory conditions may expose people to the development of obesity [26,27].

Cazettes et al. (2011) also showed that in overweight subjects, the increased concentration of fibrinogen, a marker of inflammation, was associated with an apparent elevation in the diffusion coefficient and interstitial fluid in both amygdala and right parietal cortex. These effects could damage the integrity of the microstructure of some regions of gray matter involved in the eating behavior [28]. Interestingly, various studies on obesity have established that hypothalamic inflammation is developed and can be seen weeks before the enlargement and inflammation of the adipose tissue, suggesting that the inflammation in the hypothalamus may be a noticeable factor in the appearance of the visible obese phenotype; so, it is not only a result of systemic inflammation [19,29,30]. Also, it has been reported that the prolonged secretion of TNF-α by microglia in the MHB of the mice fed a diet rich in fat or carbohydrates disrupted the function of anorexigenic neurons producing proopiomelanocortin, thus suggesting the contribution of hypothalamic microglia to the diet-induced obesity [30,31].

#### 3.1.3. Role of inflammation in the pathogenesis of neural diseases

Involvement of inflammatory cytokines in the pathogenesis of neural diseases has been revealed in various studies. In this regard, it has been shown that in depression, inflammatory cytokines such as IL-6 and TNF-α cause hypercortisolemia, which is due to the stimulation of the adrenocorticotropic hormone (ACTH) release. ACTH, in turn, induces the apoptosis of neurons and decreases the number of dendritic columns, synapses and glial cells population, causing dendritic atrophy [32]. In obesity, inflammation damages the blood vessel integrity, resulting in the infiltration of more inflammatory cells into the cerebrospinal fluid and perivascular spaces in the brain [33]. Also, the inflammatory cytokines released from the adipose tissue and entered into the bloodstream may damage cerebrovascular endothelial cells in the blood–brain barrier (BBB) [34]. In obesity, inflammation and inflammatory damage resulting from the production of IL-6 and TNF-α in the adipose tissue can be regarded as a central mechanism in the pathogenesis of Alzheimer’s by damaging glia and neurons; this is because these factors can cross the BBB and enter the central nervous system. Also, moderate inflammation caused by adiponectin can damage the BBB. As obesity is increased, the production of cortisol, estrogen, thyroid hormone, growth hormone, and insulin-like growth factor-1 (IGF-1) is increased too. These hormones, insulin, and leptin can affect the cognition, long-term potentiation (LTP) and neuroprotection in the hippocampus. Therefore, excess cortisol, probably due to excitotoxic glutamatergic neurotransmission, causes hippocampal atrophy and memory loss [13]. The regions in the brain that are affected by inflammation are shown in Table 1.

An imbalance between production and clearance of a protein named amyloid-β (Aβ) is an early step in the pathogenesis of AD. Accumulation of this protein culminates during neuronal degeneration and dementia [35]. In the brain, the amyloid deposition and other neuropathological processes that cause dementia are associated with local inflammatory events such as the secretion of interleukin, TNF-α, and other inflammatory factors [2]. Research shows that in mice, the stimulation of the immune system is associated with the deposition of the amyloid precursor protein (APP) and alteration in tau phosphorylation [36]. Besides, AD patients suffer from the high blood concentration of IL-6, TNF-α, IL-1β, transforming growth factor-beta, IL-12 and IL-8 [37].

### 3.2. Oxidative stress status in obesity

It is suggested that feeding a diet high in fat plays a potential role in decreasing the antioxidant response because it has been shown that feeding the high-fat diet in the obesity models increases protein oxidation and reduces cognitive performance in the rodents. In this line, a study conducted on male C57Bl/6 mice fed either ‘western diet’ (41% fat) or a very high-fat lard diet (60% fat) for 16 weeks showed that the latter increased adiposity, fasting blood glucose and age-related protein carbonyl (a biomarker of oxidative damage) in the hippocampus and impaired retention in the behavioral test (T-maze). This cognitive decline and oxidative stress were associated with a reduction in Nuclear Transcription Factor Erythroid 2–Related Factor (Nrf) 2 levels and Nrf2 activity [38].

#### 3.2.1. Biochemical pathways in the induction of oxidative stress in obesity

In the pentose phosphate pathway, as the main pathway for the generation of NADPH, the glucose-6-phosphate dehydrogenase (G6PD) enzyme is involved in the metabolism of glucose to ribose-5-phosphate; a decrease in its activity can result in a reduction of NADPH production, thereby promoting oxidative stress [15]. Under high glucose conditions usually observed in obese individuals with lower physical activity and overnutrition, the raised activity of protein kinase A (PKA) could increase the phosphorylation of G6PD; this, in turn, reduces the production of NADPH, subsequently causing oxidative stress [15,39]. The enzyme glutathione reductase reduces glutathione disulfide (GSSG) to an important free radical scavenger called glutathione. NADPH is needed for this activity. During obesity, overnutrition and hyperglycemia may lead to a decrease in the NADPH levels and a subsequent reduction of glutathione generation [15]. Some studies in AD have shown that the reduction of cognitive functions is correlated with GSSG [40]. Also, glutathione depletion causes the induction of apoptosis and oxidative toxicity due to the cytosolic calcium overload in the hippocampus [41]. Thioredoxin (Trx) is a redox sensor protein reducing oxidized proteins by an exchange of cysteine thioldisulfide. Based on this process, oxidized thioredoxin is reduced by NADPH [42]. In obese individuals, the higher levels of glucose and oxidative stress can up-regulate the oxidative stress mediator thioredoxin-interacting protein (TxNIP), which is an inhibitor of thioredoxin. TxNIP may contribute to the pathogenesis of AD because Aβ can induce TxNIP expression in vitro and the brains of the 5XFAD Alzheimer mice model [43]. Also, oxidative stress can increase the expression of TxNIP, which may lead to neurotoxicity [44].

#### 3.2.2. Effects of oxidative stress on the development of neurodegenerative disorders

Oxidative stress is one of the predisposing factors contributing to the damage to brain tissues and development of the central nervous system chronic inflammatory and neurodegenerative disorders, like AD, Huntington disease and Parkinson’s disease, as well as neuropsychiatric disorders such as anxiety disorders and depression [45]. Also, chronic oxidative stress in obese people may play an axial role in the onset of obesity-related neurodegenerative disorders because the resistance of the brain against oxidative stress is lower than that of other organs [46]. Oxidative stress causes neurodegeneration by increasing ROS and lowering the antioxidant capacity [47]. In the brains of the patients with AD, the activity of antioxidant enzymes SOD, catalase, glutathione Peroxidase (GPx) and glutathione reductase is reduced [48,49].

In obesity, the production of ROS may cause oxidative damage to cellular molecules and cytoskeleton, contributing to the BBB. It has been suggested that in obesity, up-regulation of cytokines and increased oxidative stress may damage the BBB through the disruption of tight junctions and augmentation of neuroinflammatory responses [50]. Oxidative stress may induce apoptosis and neurodegeneration in the brain [51]. Also, some studies suggest oxidative stress may be among underlying causes involved in the development of Parkinson’s disease. For example, in Parkinson’s disease, the excess accumulation of neurotoxic α-synuclein occurs and oxidative stress promotes α-synuclein aggregation in dopaminergic neurons. α-synuclein, in turn, produces more intracellular ROS [52].

#### 3.2.3. Vulnerability of the hippocampus and amygdala to oxidative stress

Cerebellar granule cells, the hippocampus and amygdala are involved in the behavioral and cognitive deficits; these areas are very vulnerable to oxidative stress. In the hippocampus, the dentate gyrus plays an important role in learning and memory functions; the ventral hippocampus is involved in anxiety and depression. Oxidative stress, through alteration in the cell proliferation, neurogenesis, capacity remodeling, and changes in the structural plasticity, can impair the biochemical integrity of the hippocampus and the amygdala, as well as their functions; as a consequence, it may disturb the normal synaptic neurotransmission [45]. The raised concentrations of ROS affect the long-term potentiation (LTP), synaptic signaling and brain plasticity [53]. The increase in the ROS production could affect LTP by changing the N-methyl-d-aspartate (NMDA)-dependent calcium influx. In the CA1 synapses of the hippocampus, superoxide acts as a signal in LTP and chronic oxidative stress is a main reason for the decreased LTP response [54]. Also, obesity-related oxidative stress may impair superoxide signaling in the hippocampal neurons and the subsequent dysfunction of synapses may be a reason for the impaired cognitive function in obesity. Studies have also shown that the increased concentrations of the advanced glycation end products (AGEs) in the circulation and brain are associated with cognitive dysfunctions in the old subjects with AD [55]. AGEs facilitate the formation of amyloid plaques and increase the cytotoxicity of Aβ. Also, the expression of the AGE receptor, called RAGE, is increased by AGEs, and RAGE may be a receptor for Aβ [21].

Oxidative stress may cause amygdalar hyperactivity and dendritic shrinking, which could further exacerbate synaptic impairments by interrupting the hippocampus-amygdala projections. Also, free radicals may oxidize the extracellular sites of glutamatergic NMDA receptors, subsequently attenuating LTP and synaptic neurotransmission; this, in turn, may result in behavioral and cognitive impairment [45]. Also, in the brain, the increased levels of free radicals may cause damage to cellular structures such as membranes, DNA and cellular proteins; it may change the activity of transcriptional factors such as NF-κB, thereby resulting in chronic inflammation and cell apoptosis [56]. Although one study in male C57BL/6 mice showed that the induction of obesity by high-fat diet during a 7–8 month period increased the levels of ROS parameters total ROS, superoxide and peroxynitrite within the cerebral cortex, while decreasing the GPx activity in the cerebral cortex, the serum concentrations of 8-Isoprostane, a biomarker of peripheral oxidative stress, were not significantly altered; the results also showed that the elevation of ROS in the brain were correlated with adiposity, but the increase in the ROS was not correlated with a cognitive decline in these models of obesity [57]. To summarize, the experimental findings, as shown above, suggest that in obese subjects, the oxidative stress may be an axial mechanism for the development of various brain disorders and neural diseases such as AD and dementia (Table 2).

### 3.3. The influence of IR on the brain functions

IR means a decrease in the ability of the insulin receptor to respond to this hormone; as a result, pancreatic beta cells begin to produce more insulin, leading to hyperinsulinemia. Inflammatory factors such as TNF-α, free fatty acid (FFA) and their downstream factors such as amino-terminal c-Jun kinases (JNK) are the most important linking factors between obesity and IR [58].

About 80% of Alzheimer’s patients have T2DM or hyperglycemia. Animal models of diabetes also show an association between abnormal glucose metabolism, memory impairment and synaptic plasticity disruption, suggesting that hyperglycemia can impair cognitive functions [58].

Research also shows that the presence of the excess insulin in the brain is associated with a reduction of cognitive impairment [58]; and hyper-insulinemia is a predisposing factor in the development of dementia, defects of insulin signaling, and impairment of glucose metabolism in the brain. It is suggested that hyperinsulinemia is related to AD; so, the use of insulin in the AD patients improves the process of memory formation [59,60]. Various studies have shown the involvement of insulin in the growth of neurite, regulation of secretion, uptake of catecholamines, trafficking of ligand-containing ion channels, and modulation of synaptic flexibility through NMDA and PI3K/AKT. Also, insulin plays a crucial role in the formation and maintenance of excitatory synapses and neural stem cells activation [15,59]. This evidence suggests that the obesity-induced IR may have a detrimental effect on brain functions.

#### 3.3.1. The mechanisms linking the obesity associated IR to the brain impairments

In the chronic peripheral IR and hyperinsulinemia conditions, the decrease in the insulin transport across the BBB is seen; this, in turn, decreases the insulin concentration and its activity in the brain. Various studies of AD patients have shown that in their central nervous system, markers of brain insulin-signaling are decreased [58,61]. Also, in the streptozotocin-induced diabetic mouse model, the decreased brain insulin signaling was associated with the increased phosphorylation of tau protein and the increased Aβ levels [62]. Besides this, using a diet high in fat causes IR in the hypothalamus and telencephalon, which is correlated to the defects of synaptic plasticity, synaptic integrity and cognitive behaviors [59,60]. On the other hand, obesity and T2DM are two predicting factors for cardiovascular diseases that may impair cognitive performance by reducing the cerebral blood flow in the cerebrum [2]. Moreover, in obesity, the transport of insulin across the BBB is decreased [63,64].

In one study, a mouse model of obesity was developed by treating mice with a diet high in fat and sugar (fructose and sucrose); the results showed that this diet reduced the expression of glucose transporter (GLUT) 1, GLUT3, and the insulin-degrading enzyme in the brain of the mice models, as compared to the controls. Considering the role of the insulin-degrading enzyme in the degradation of Aβ, their results imply that the degradation and clearance of Aβ were decreased [59]. The Aβ oligomers toxicity directly induces neuronal resistance to insulin and inhibits LTP [58].

On the other hand, the chronic overactivation of AKT by IR may lead to the hyperphosphorylation of AMPK Ser485 and the subsequent inhibition of Tau dephosphorylation. Hyperphosphorilation of Tau decreases its solubility; therefore, it cannot assemble tubulins for the formation of microtubules and vesicular transport, resulting in neurodegeneration [59]. In a mice model of obesity, AKT and AMPK Ser485 were hyperactivated in comparison to controls [59]. As mentioned previously, activation of PKA can impair synaptic activity through phosphorylation of the tau protein [15].

One membrane-associated scaffolding protein, called PSD-95, is an important regulator of synaptic strength. The results of a study showed that PSD-95 gene expression was decreased in obese mice, as compared to the control ones [59]. Ser phosphorylation of insulin receptor substrate-1 (IRS-1) resulted in the inhibition of the IRS-1 activity and the subsequent IRS-1 degradation. Another study also showed that the high-fat and sugar diet induced IR in the neurons by decreasing the tyrosine phosphorylation of IRS-1 and increasing IRS-1 serine phosphorylation. Further, these were associated with the activation of stress (JNK and CHOP) and inflammatory pathways (NFκB) in the brains of animals [59]. Also, oxidative stress plays a central role in IR [65]. Oxidative stress and inflammatory cytokines cause the increase in the IRS-1 and Akt serine phosphorylation, leading to the decrease in the insulin receptor expression and tyrosine phosphorylation, which can augment IR [58]. Therefore, in obesity, oxidative stress and inflammation can induce the brain IR. To summarize, during obesity, the IR induced by oxidative stress and inflammation may be one of the important factors which could impair the hippocampal functions, thus contributing to the development of neurodegenerative diseases like AD.

### 3.4. The obesity induced-hypertension

Various clinical and animal studies have reported that obesity, especially visceral obesity, is among factors contributing to developing hypertension and cardiovascular diseases [66,67]. The obese people are more likely to have high blood pressure and arterial stiffness; it has been shown that the systolic and diastolic blood pressure, and pulse wave velocity (PWV) in the obese subjects are increased, in comparison to the normal weight people. Obesity increases the intravascular inflammation, altering the endothelial function, increasing the thickness of the intima-media and reducing the lumen diameter of arteries [68]. Moreover, there is much evidence suggesting that obesity, through different mechanisms such as activation of the renin-angiotensin-aldosterone system, the raised sympathetic activity, IR, leptin resistance, the increased coagulation activity, endothelial dysfunction and enhanced renal sodium reabsorption, raises the blood pressure [69].

#### 3.4.1. The effects of hypertension induced by obesity on the brain

One of the mechanisms suggested for the association between obesity and the increased risk of dementia is hypertension; some studies suggest that obese people are disposed to hypertension and hypertensive people are disposed to dementia; hypertension is an independent risk factor for AD [66,67,70]. Various epidemiological studies show that high blood pressure and cerebrovascular diseases in middle age are associated with cognitive defects and dementia, especially AD and vascular dementia [71,72].

The evidence obtained from magnetic resonance imaging suggests that high blood pressure can affect the brain; however, the underlying mechanisms of the effects of high blood pressure on the brain and the association of high blood pressure with AD are unclear [73]. In this regard, a study of 3735 Japanese-American men in Hawaii found that the increased systolic blood pressure (BP) at the middle age might be a predictor of a future cognitive decline. Their findings also showed that a 10 mm Hg rise in the systolic BP could be associated with the decreased cognitive function [74]. Also, a study of a sample of Japanese-American men born between 1900 and 1919 examined the association between middle-aged BP and the late hippocampal atrophy, showing that people with high systolic BP were at risk for hippocampal atrophy [14].

Also, one study examined the relationship between BMI and the higher systolic and diastolic blood pressure and the decreased motor function and agility, and executive performance tests; the obtained results showed that there was a negative relationship between total and central obesity and cognitive function [75]. In addition, the MRI studies have shown that hypertensive people have damaged brain white matter and periventricular white matter [76]. Also, in these patients, the alteration in the vascular structure and vascular tone could cause tissue hypoxia and increase the blood–brain barrier permeability [77].

#### 3.4.2. The mechanisms underlying the relationship between the effects of hypertension on the brain

In the brains of the patients with vascular dementia, the infiltration of plasma constituents and inflammatory cells into the arterial walls and the surrounding vascular tissue can lead to atherosclerosis, fibrinoid necrosis, lipohyalinosis, endothelial dysfunction, and BBB breakdown. Endothelial dysfunction and inflammation may thicken the arterial wall, thus increasing the risk of ischemia. Moreover, in these cases, the ability to expand the damaged arterial walls is limited [5].

The cerebral blood flow is synchronized with cerebral activity. Hypertension through the improper regulation of vasoactive mediators including nitric oxide (NO) and endothelin-1, induction of oxidative stress, blood vessels restructuring, and inadequate brain readjustmentmay affect this process, leading to vascular dementia [78]. Under hypertensive conditions, the inflammatory response induced by neuroglial cells and the stimulation of metalloproteinase 9 production by oligodendrocytes can have destructive effects on the BBB; also, in the arteries of this region, the fibrohyaline deposition and atherosclerosis occur [79]. In some cases, there is a loss of vascular wall integrity and a large amount of hyaline deposit [80]. Besides this, evidence obtained from models linking hypertension to the cognitive function suggests that silent clinical stroke, metabolic imbalance, the altered distribution of cerebral blood flow, demyelination or microinfarction of the white matter of the brain may be among the pathways linking high blood pressure to the risk of cognitive impairment; many of these conditions are seen in obesity [81]. To sum it up, in the obese subjects, the high blood pressure may increase the risk of cognitive impairment and dementia without stroke [73].

## 4. Conclusions

By considering the factors discussed previously, it is likely that the consequences of obesity on brain functions are mediated by oxidative stress, inflammation and hypertension; these factors, when associated with obesity, make people vulnerable to stroke, dementia and AD. Therefore, management of obesity by different approaches like caloric restriction, regular physical activity and pharmacological treatments may be particularly important for the prevention of neural disorders such as dementia and AD. Considering the growing obese and overweight population worldwide, it is likely that the health care systems may be facing subtle executive dysfunction and cognitive decline in the future. Awareness obtained from studies of the brain complications associated with obesity and/or a high-fat diet can result in the development of new ways to postpone cognitive dysfunction. However, a complex set of reciprocal actions are involved in the effect of obesity on brain health; so, more research is needed to describe the exact link between the effects of obesity and overweight and impaired brain functions.

## Figures and Tables

**Table 1:** The brain areas influenced by inflammation during obesity and/or use of a diet high in fat.

The brain regions influenced by inflammation	Reference
Hypothalamus, amygdala, hippocampus, brain cortex and cerebellum	[23,24]
Blood vessel integrity and perivascular spaces in the brain	[33]
Amygdala, right parietal cortex and the areas in the gray matter involved in the eating behavior	[28]
The hypothalamic area involved in the control of the feeding behavior	[25]

**Table 2:** Summary of oxidative stress effects on the brain during obesity^a^.

The effects	Reference
Impairment of biochemical integrity in the hippocampus and amygdala; alteration of structural plasticity, remodeling capacity, and synaptic neurotransmission in the amygdala; impairment of cell proliferation, neurogenesis in the amygdala	[45]
Influence on LTP and synaptic signaling	[53]
Damage to the cell membrane, DNA and proteins; induction of chronic inflammation and cell apoptosis, and neurodegeneration	[51,56]
Hyperactivity of the amygdala and dendritic shrinking; synaptic impairment in the amygdala	[45]
Oxidation of NMDA receptors and LTP and synaptic neurotransmission in the amygdala	[45]
Damage to the BBB by the disruption of tight junctions and augmentation of neuroinflammatory responses	[50]
Increase in the NMDA-dependent Calcium influx and change in LTP	[54]
Increase in AGEs in the blood and brain that, in turn, facilitates the formation of amyloid plaques and increases the cytotoxicity of Aβ	[21]
Glutathione depletion causes cytosolic calcium overload, inducing apoptosis and oxidative toxicity in the hippocampus	[41]
The up-regulation of TxNIP, which may cause an increase in the oxidized proteins and neural death	[42,43]
Promotion of neurotoxic α-synuclein aggregation in dopaminergic neurons involved in the development of Parkinson’s disease	[52]

a LTP - Long-term Potentiation, NMDA - N-Methyl-d-Aspartate, BBB - Blood–Brain-Barrier, AGEs - Advanced Glycation End Products, Aβ - Amyloid-β, TxNIP - Thioredoxin-interacting Protein.

## References

[1] Anand SS Hawkes C De Souza RJ Mente A Dehghan M Nugent R Food consumption and its impact on cardiovascular disease: importance of solutions focused on the globalized food system: a report from the workshop convened by the World Heart Federation J Am Coll 2015 66 14 1590 614 10.1016/j.jacc.2015.07.050 PMC4597475 26429085

[2] Heiss C Goldberg L Associations among visceral obesity, type 2 diabetes, and dementia J Obes Eat Disord 2016 2 2 1 5

[3] Marseglia L Manti S D’Angelo G Nicotera A Parisi E Di Rosa G Oxidative stress in obesity: a critical component in human diseases Int J Mol Sci 2015 16 1 378 400 10.3390/ijms16010378 PMC4307252 25548896

[4] Cheke LG Bonnici HM Clayton NS Simons JS Obesity and insulin resistance are associated with reduced activity in core memory regions of the brain Neuropsychologia 2017 96 137 49 28093279 10.1016/j.neuropsychologia.2017.01.013 PMC5317178

[5] Appleton JP Scutt P Sprigg N Bath PM Hyper-cholesterolaemia and vascular dementia Clin Sci (Lond) 2017 131 14 1561 78 28667059 10.1042/CS20160382

[6] Luchsinger JA Tang M-X Shea S Mayeux R Caloric intake and the risk of Alzheimer disease Arch Neurol 2002 59 8 1258 63 12164721 10.1001/archneur.59.8.1258

[7] Gunstad J Paul RH Cohen RA Tate DF Spitznagel MB Grieve S Relationship between body mass index and brain volume in healthy adults Int J Neurosci 2008 118 11 1582 93 18853335 10.1080/00207450701392282

[8] Pannacciulli N Del Parigi A Chen K Le DSN Reiman EM Tataranni PA Brain abnormalities in human obesity: a voxel-based morphometric study Neuroimage 2006 31 4 1419 25 16545583 10.1016/j.neuroimage.2006.01.047

[9] Taki Y Kinomura S Sato K Inoue K Goto R Okada K Relationship between body mass index and gray matter volume in 1,428 healthy individuals Obesity 2008 16 1 119 24 18223623 10.1038/oby.2007.4

[10] Jagust W Harvey D Mungas D Haan M Central obesity and the aging brain Arch Neurol 2005 62 10 1545 8 16216937 10.1001/archneur.62.10.1545

[11] Whitmer R Gustafson D Barrett-Connor E Haan M Gunderson E Yaffe K Central obesity and increased risk of dementia more than three decades later Neurology 2008 71 14 1057 64 18367704 10.1212/01.wnl.0000306313.89165.ef

[12] Xu W Atti A Gatz M Pedersen N Johansson B Fratiglioni L Midlife overweight and obesity increase late-life dementia risk: a population-based twin study Neurology 2011 76 18 1568 74 21536637 10.1212/WNL.0b013e3182190d09 PMC3100125

[13] Profenno LA Porsteinsson AP Faraone SV Meta-analysis of Alzheimer’s disease risk with obesity, diabetes, and related disorders Biol Psychiatr 2010 67 6 505 12 10.1016/j.biopsych.2009.02.013 19358976

[14] Korf ES White LR Scheltens P Launer LJ Midlife blood pressure and the risk of hippocampal atrophy: the Honolulu Asia Aging Study Hypertension 2004 44 1 29 34 15159381 10.1161/01.HYP.0000132475.32317.bb

[15] Verdile G Keane KN Cruzat VF Medic S Sabale M Rowles J Inflammation and oxidative stress: the molecular connectivity between insulin resistance, obesity, and Alzheimer’s disease Mediat Inflamm 2015 2015 10.1155/2015/105828 PMC4674598 26693205

[16] Widya RL de Roos A Trompet S de Craen AJ Westendorp RG Smit JW PROSPER Study Group Increased amygdalar and hippocampal volumes in elderly obese individuals with or at risk of cardiovascular disease Am J Clin Nutr 2011 93 6 1190 5 21450935 10.3945/ajcn.110.006304

[17] Ronan L Alexander-Bloch AF Wagstyl K Farooqi S Brayne C Tyler LK Obesity associated with increased brain age from midlife Neurobiol Aging 2016 47 63 70 27562529 10.1016/j.neurobiolaging.2016.07.010 PMC5082766

[18] Carnell S Gibson C Benson L Ochner C Geliebter A Neuroimaging and obesity: current knowledge and future directions Obes Rev 2012 13 1 43 56 21902800 10.1111/j.1467-789X.2011.00927.x PMC3241905

[19] Thaler JP Yi C-X Schur EA Guyenet SJ Hwang BH Dietrich MO Obesity is associated with hypothalamic injury in rodents and humans J Clin Invest 2012 122 1 153 62 22201683 10.1172/JCI59660 PMC3248304

[20] Trentham-Dietz A Newcomb PA Nichols HB Hampton JM Breast cancer risk factors and second primary malignancies among women with breast cancer Breast Cancer Res Treat 2007 105 2 195 207 17186360 10.1007/s10549-006-9446-y

[21] Pugazhenthi S Qin L Reddy PH Common neurodegenerative pathways in obesity, diabetes, and Alzheimer’s disease Biochim Biophys Acta (BBA) - Mol Basis Dis 2017 1863 5 1037 45 10.1016/j.bbadis.2016.04.017 PMC5344771 27156888

[22] Qin L Bouchard R Pugazhenthi S Regulation of cyclic AMP response element-binding protein during neuroglial interactions J Neurochem 2016 136 5 918 30 26677139 10.1111/jnc.13497

[23] Guillemot-Legris O Muccioli GG Obesity-induced neuroinflammation: beyond the hypothalamus Trends Neurosci 2017 40 4 237 53 28318543 10.1016/j.tins.2017.02.005

[24] Tapia-González S García-Segura LM Tena-Sempere M Frago L Castellano J Fuente-Martín E Activation of microglia in specific hypothalamic nuclei and the cerebellum of adult rats exposed to neonatal overnutrition J Neuroendocrinol 2011 23 4 365 70 21314736 10.1111/j.1365-2826.2011.02113.x

[25] Velloso LA Araújo EP de Souza CT Diet-induced inflammation of the hypothalamus in obesity Neuroimmunomodulation 2008 15 3 189 93 18781083 10.1159/000153423

[26] Cansell C Stobbe K Sanchez C Le Thuc O Mosser CA Ben Fradj S Dietary fat exacerbates postprandial hypothalamic inflammation involving glial fibrillary acidic protein-positive cells and microglia in male mice Glia 2020 69 1 42 60 32659044 10.1002/glia.23882

[27] Balland E Cowley M Short-term high-fat diet increases the presence of astrocytes in the hypothalamus of C57 BL 6 mice without altering leptin sensitivity J Neurol 2017 29 10 e12504 10.1111/jne.12504 28699230

[28] Cazettes F Cohen JI Yau PL Talbot H Convit A Obesity-mediated inflammation may damage the brain circuit that regulates food intake Brain Res 2011 1373 101 9 21146506 10.1016/j.brainres.2010.12.008 PMC3026911

[29] Gao Y Ottaway N Schriever SC Legutko B García-Cáceres C de la Fuente E Hormones and diet, but not body weight, control hypothalamic microglial activity Glia 2014 62 1 17 25 24166765 10.1002/glia.22580 PMC4213950

[30] Macedo F dos Santos LS Glezer I da Cunha FM Brain innate immune response in diet-induced obesity as a paradigm for metabolic influence on inflammatory signaling Front Neurosci 2019 13 342 31068773 10.3389/fnins.2019.00342 PMC6491681

[31] Yi C-X Walter M Gao Y Pitra S Legutko B Kälin S TNFα drives mitochondrial stress in POMC neurons in obesity Nat Commun 2017 8 1 1 9 28489068 10.1038/ncomms15143 PMC5436136

[32] McKay MS Zakzanis KK The impact of treatment on HPA axis activity in unipolar major depression J Psychiatr Res 2010 44 3 183 92 19747693 10.1016/j.jpsychires.2009.07.012

[33] Man S Ubogu EE Ransohoff RM Inflammatory cell migration into the central nervous system: a few new twists on an old tale Brain Pathol 2007 17 2 243 50 17388955 10.1111/j.1750-3639.2007.00067.x PMC8095646

[34] Tarantini S Valcarcel-Ares MN Yabluchanskiy A Tucsek Z Hertelendy P Kiss T Nrf2 deficiency exacerbates obesity-induced oxidative stress, neurovascular dysfunction, blood–brain barrier disruption, neuroinflammation, amyloidogenic gene expression, and cognitive decline in mice, mimicking the aging phenotype J Gerontol 2018 73 7 853 63 10.1093/gerona/glx177 PMC6001893 29905772

[35] Hardy J Selkoe DJ The amyloid hypothesis of Alzheimer’s disease: progress and problems on the road to therapeutics Science 2002 297 5580 353 6 12130773 10.1126/science.1072994

[36] Krstic D Madhusudan A Doehner J Vogel P Notter T Imhof C Systemic immune challenges trigger and drive Alzheimer-like neuropathology in mice J Neuroinflammation 2012 9 1 151 22747753 10.1186/1742-2094-9-151 PMC3483167

[37] Swardfager W Lanctôt K Rothenburg L Wong A Cappell J Herrmann N A meta-analysis of cytokines in Alzheimer’s disease Biol Psychiatr 2010 68 10 930 41 10.1016/j.biopsych.2010.06.012 20692646

[38] Morrison CD Pistell PJ Ingram DK Johnson WD Liu Y Fernandez-Kim SO High fat diet increases hippocampal oxidative stress and cognitive impairment in aged mice: implications for decreased Nrf2 signaling J Neurochem 2010 114 6 1581 9 20557430 10.1111/j.1471-4159.2010.06865.x PMC2945419

[39] Xu Y Osborne BW Stanton RC Diabetes causes inhibition of glucose-6-phosphate dehydrogenase via activation of PKA, which contributes to oxidative stress in rat kidney cortex Am J Physiol Ren Physiol 2005 289 5 F1040 7 10.1152/ajprenal.00076.2005 15956780

[40] Cristalli DO Arnal N Marra FA de Alaniz MJ Marra CA Peripheral markers in neurodegenerative patients and their first-degree relatives J Neurol Sci 2012 314 1–2 48 56 22113180 10.1016/j.jns.2011.11.001

[41] Övey I Naziroğlu M Homocysteine and cytosolic GSH depletion induce apoptosis and oxidative toxicity through cytosolic calcium overload in the hippocampus of aged mice: involvement of TRPM2 and TRPV1 channels Neuroscience 2015 284 225 33 25305668 10.1016/j.neuroscience.2014.09.078

[42] Minn AH Hafele C Shalev A Thioredoxin-interacting protein is stimulated by glucose through a carbohydrate response element and induces β-cell apoptosis Endocrinology 2005 146 5 2397 405 15705778 10.1210/en.2004-1378

[43] Gouget T Djelloul M Boucraut J Weinhard L Baranger K Rivera S TXNIP, the major player in insulin resistance, is early over-expressed in the brain of the 5XFAD Alzheimer’s mice model and is induced by Aβ in vitro: emerging role of TXNIP and inflammation in Alzheimer’s Disease progression Alzheimers Dement 2011 7 4 S684

[44] Kim GS Jung JE Narasimhan P Sakata H Chan PH Induction of thioredoxin-interacting protein is mediated by oxidative stress, calcium, and glucose after brain injury in mice Neurobiol Dis 2012 46 2 440 9 22366181 10.1016/j.nbd.2012.02.008 PMC3323710

[45] Salim S Oxidative stress and the central nervous system J Pharmacol Exp Therapeut 2017 360 1 201 5 10.1124/jpet.116.237503 PMC5193071 27754930

[46] Roh H-T Cho S-Y So W-Y Obesity promotes oxidative stress and exacerbates blood-brain barrier disruption after high-intensity exercise J Sport Health Sci 2017 6 2 225 30 30356585 10.1016/j.jshs.2016.06.005 PMC6188985

[47] Mariani E Polidori M Cherubini A Mecocci P Oxidative stress in brain aging, neurodegenerative and vascular diseases: an overview J Chromatogr B 2005 827 1 65 75 10.1016/j.jchromb.2005.04.023 16183338

[48] Pappolla M Omar R Kim K Robakis N Immunohistochemical evidence of oxidative [corrected] stress in Alzheimer’s disease Am J Pathol 1992 140 3 621 1372157 PMC1886174

[49] Zemlan FP Thienhaus OJ Bosmann HB Superoxide dismutase activity in Alzheimer’s disease: possible mechanism for paired helical filament formation Brain Res 1989 476 1 160 2 2521568 10.1016/0006-8993(89)91550-3

[50] Tucsek Z Toth P Sosnowska D Gautam T Mitschelen M Koller A Obesity in aging exacerbates blood–brain barrier disruption, neuroinflammation, and oxidative stress in the mouse hippocampus: effects on expression of genes involved in beta-amyloid generation and Alzheimer’s disease J Gerontol 2013 69 10 1212 26 10.1093/gerona/glt177 PMC4172034 24269929

[51] Reynolds A Laurie C Mosley RL Gendelman HE Oxidative stress and the pathogenesis of neurodegenerative disorders Int Rev Neurobiol 2007 82 297 325 17678968 10.1016/S0074-7742(07)82016-2

[52] Xiang W Schlachetzki JC Helling S Bussmann JC Schäffer TE Berlingh of M Oxidative stress-induced posttranslational modifications of alpha-synuclein: specific modification of alpha-synuclein by 4-hydroxy-2-nonenal increases dopaminergic toxicity Mol Cell Neurosci 2013 54 71 83 23369945 10.1016/j.mcn.2013.01.004

[53] Knapp LT Klann E Role of reactive oxygen species in hippocampal long-term potentiation: contributory or inhibitory? J Neurosci Res 2002 70 1 1 7 12237859 10.1002/jnr.10371

[54] Farr SA Yamada KA Butterfield DA Abdul HM Xu L Miller NE Obesity and hypertriglyceridemia produce cognitive impairment Endocrinology 2008 149 5 2628 36 18276751 10.1210/en.2007-1722 PMC2329289

[55] Beeri MS Moshier E Schmeidler J Godbold J Uribarri J Reddy S Serum concentration of an inflammatory glycotoxin, methylglyoxal, is associated with increased cognitive decline in elderly individuals Mech Ageing Dev 2011 132 11–12 583 7 22079406 10.1016/j.mad.2011.10.007 PMC3243767

[56] Newsholme P Morgan D Rebelato E Oliveira-Emilio H Procopio J Curi R Insights into the critical role of NADPH oxidase (s) in the normal and dysregulated pancreatic beta cell Diabetologia 2009 52 12 2489 98 19809798 10.1007/s00125-009-1536-z

[57] Freeman LR Zhang L Nair A Dasuri K Francis J Fernandez-Kim S-O Obesity increases cerebrocortical reactive oxygen species and impairs brainfunction Free Radic Biol Med 2013 56 226 33 23116605 10.1016/j.freeradbiomed.2012.08.577 PMC4038352

[58] Zhao W-Q Townsend M Insulin resistance and amyloidogenesis as common molecular foundation for type 2 diabetes and Alzheimer’s disease Biochim Biophys Acta 2009 1792 5 482 96 19026743 10.1016/j.bbadis.2008.10.014

[59] Kothari V Luo Y Tornabene T O’Neill AM Greene MW Geetha T High fat diet induces brain insulin resistance and cognitive impairment in mice Biochim Biophys Acta (BBA) - Mol Basis Dis 2017 1863 2 499 508 10.1016/j.bbadis.2016.10.006 27771511

[60] Liu Z Patil IY Jiang T Sancheti H Walsh JP Stiles BL High-fat diet induces hepatic insulin resistance and impairment of synaptic plasticity PloS One 2015 10 5 e0128274 26023930 10.1371/journal.pone.0128274 PMC4449222

[61] Craft S The role of metabolic disorders in Alzheimer disease and vascular dementia: two roads converged Arch Neurol 2009 66 3 300 5 19273747 10.1001/archneurol.2009.27 PMC2717716

[62] Jolivalt C Lee C Beiswenger K Smith J Orlov M Torrance M Defective insulin signaling pathway and increased glycogen synthase kinase-3 activity in the brain of diabetic mice: parallels with Alzheimer’s disease and correction by insulin J Neurosci Res 2008 86 15 3265 74 18627032 10.1002/jnr.21787 PMC4937800

[63] Kaiyala KJ Prigeon RL Kahn SE Woods SC Schwartz MW Obesity induced by a high-fat diet is associated with reduced brain insulin transport in dogs Diabetes 2000 49 9 1525 33 10969837 10.2337/diabetes.49.9.1525

[64] Rhea EM Salameh TS Logsdon AF Hanson AJ Erickson MA Banks WA Blood-brain barriers in obesity AAPS J 2017 19 4 921 30 28397097 10.1208/s12248-017-0079-3 PMC5972029

[65] Aroor AR DeMarco VG Oxidative stress and obesity: the chicken or the egg? Diabetes 2014 63 7 2216 8 24962921 10.2337/db14-0424

[66] Hall JE The kidney, hypertension, and obesity Hypertension 2003 41 3 625 33 12623970 10.1161/01.HYP.0000052314.95497.78

[67] Sironi AM Gastaldelli A Mari A Ciociaro D Postano V Buzzigoli E Visceral fat in hypertension: influence on insulin resistance and β-cell function Hypertension 2004 44 2 127 33 15262911 10.1161/01.HYP.0000137982.10191.0a

[68] Son W-M Kim D-Y Kim Y-S Ha M-S Effect of obesity on blood pressure and arterial stiffness in middle-aged Korean women Osong Publ Health Res Perspect 2017 8 6 369 10.24171/j.phrp.2017.8.6.02 PMC5749481 29354393

[69] Narkiewicz K Obesity and hypertension—the issue is more complex than we thought Nephrol Dial Transplant 2006 21 2 264 7 16311261 10.1093/ndt/gfi290

[70] Capizzano AA Acion L Bekinschtein T Furman M Gomila H Martinez A White matter hyperintensities are significantly associated with cortical atrophy in Alzheimer’s disease J Neurol Neurosurg Psychiatry 2004 75 6 822 7 15145992 10.1136/jnnp.2003.019273 PMC1739041

[71] Reijmer YD van den Berg E Dekker JM Nijpels G Stehouwer CD Kappelle LJ Development of vascular risk factors over 15 Years in relation to cognition: the H oorn study J Am Geriatr Soc 2012 60 8 1426 33 22861348 10.1111/j.1532-5415.2012.04081.x

[72] Baumgart M Snyder HM Carrillo MC Fazio S Kim H Johns H Summary of the evidence on modifiable risk factors for cognitive decline and dementia: a population-based perspective Alzheimers Dement 2015 11 6 718 26 26045020 10.1016/j.jalz.2015.05.016

[73] Tzourio C Hypertension, cognitive decline, and dementia: an epidemiological perspective Dialogues Clin Neurosci 2007 9 1 61 17506226 10.31887/DCNS.2007.9.1/ctzourio PMC3181842

[74] Launer LJ Masaki K Petrovitch H Foley D Havlik RJ The association between midlife blood pressure levels and late-life cognitive function: the Honolulu-Asia Aging Study JAMA 1995 274 23 1846 51 7500533

[75] Waldstein S Katzel L Interactive relations of central versus total obesity and blood pressure to cognitive function Int J Obes 2006 30 1 201 7 10.1038/sj.ijo.0803114 16231030

[76] Jiménez-Balado J Riba-Llena I Abril O Garde E Penalba A Ostos E Cognitive impact of cerebral small vessel disease changes in patients with hypertension Hypertension 2019 73 2 342 9 30606062 10.1161/HYPERTENSIONAHA.118.12090

[77] Ueno M Tomimoto H Akiguchi I Wakita H Sakamoto H Blood–brain barrier disruption in white matter lesions in a rat model of chronic cerebral hypoperfusion J Cerebr Blood Flow Metabol 2002 22 1 97 104 10.1097/00004647-200201000-00012 11807399

[78] Iadecola C Davisson RL Hypertension and cerebrovascular dysfunction Cell Metab 2008 7 6 476 84 18522829 10.1016/j.cmet.2008.03.010 PMC2475602

[79] Pantoni L Cerebral small vessel disease: from pathogenesis and clinical characteristics to therapeutic challenges Lancet Neurol 2010 9 7 689 701 20610345 10.1016/S1474-4422(10)70104-6

[80] Wardlaw JM Smith C Dichgans M Mechanisms of sporadic cerebral small vessel disease: insights from neuroimaging Lancet Neurol 2013 12 5 483 97 23602162 10.1016/S1474-4422(13)70060-7 PMC3836247

[81] Knopman DS Mosley TH Catellier DJ Sharrett AR Cardiovascular risk factors and cerebral atrophy in a middle-aged cohort Neurology 2005 65 6 876 81 16186527 10.1212/01.wnl.0000176074.09733.a8

